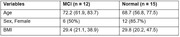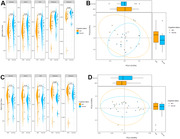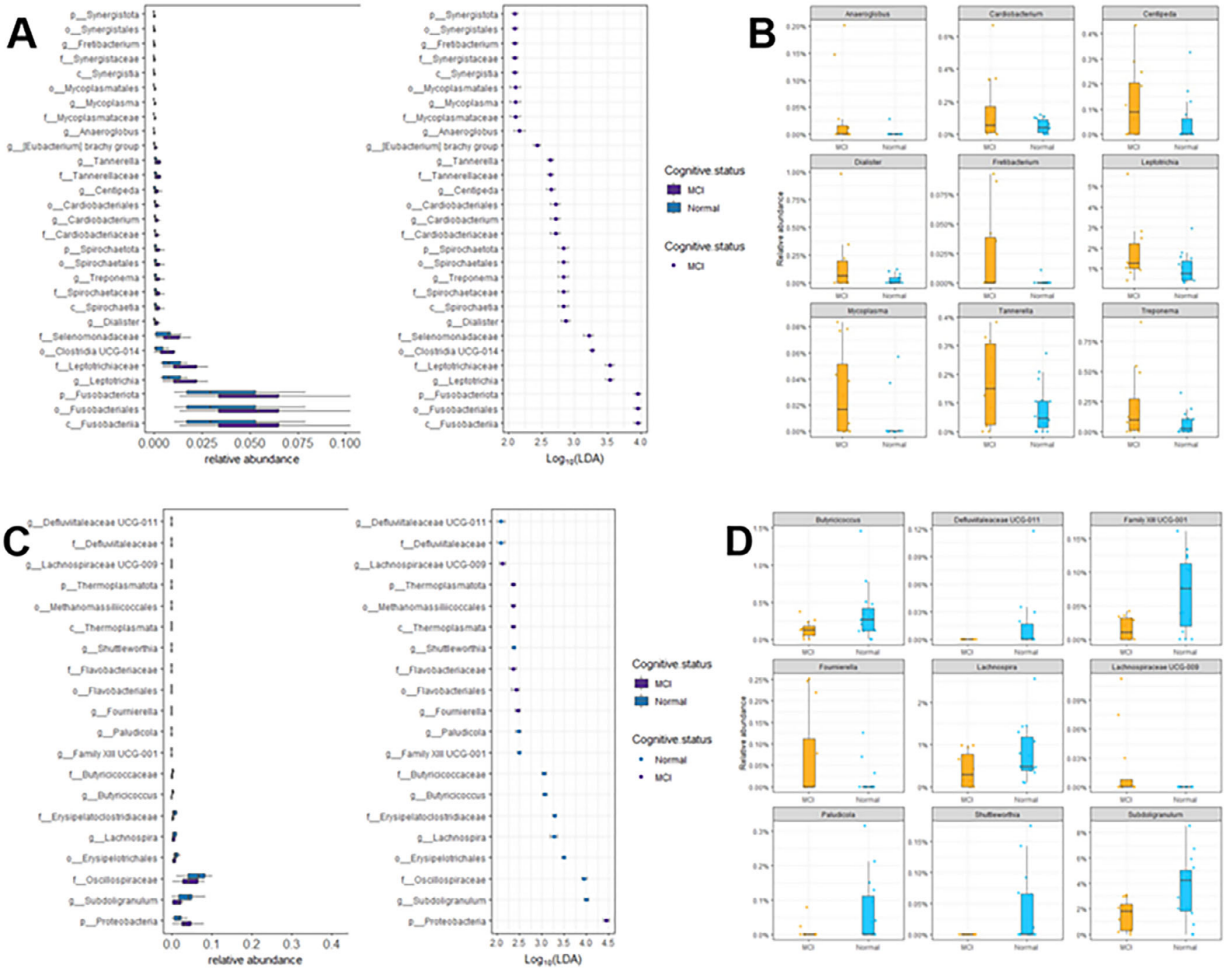# Differential Patterns of Gut and Oral Microbiomes in Patients with Mild‐cognitive Impairment

**DOI:** 10.1002/alz.089090

**Published:** 2025-01-03

**Authors:** Yannick Joel Wadop Ngouongo, Julia J Mathews, Jazmyn A Muhammad, Rosa P. Pirela‐Mavarez, Michael C Mahaney, Gladys E. Maestre, Sudha Seshadri, Tiffany F. Kautz, Bernard Fongang

**Affiliations:** ^1^ Glenn Biggs Institute for Alzheimer’s & Neurodegenerative Diseases, The University of Texas Health Science Center at San Antonio, San Antonio, TX USA; ^2^ Department of Neurosciences and Department of Human Genetics, University of Texas Rio Grande Valley School of Medicine, Brownsville, TX USA; ^3^ University of Texas Rio Grande Valley School of Medicine, Brownsville, TX USA; ^4^ Glenn Biggs Institute for Alzheimer’s & Neurodegenerative Diseases, San Antonio, TX USA; ^5^ Department of Neurology, UT Health San Antonio, 7703 Floyd Curl Drive, San Antonio, TX USA; ^6^ Framingham Heart Study, Framingham, MA USA; ^7^ Department of Neurology, Boston University School of Medicine, Boston, MA USA; ^8^ Glenn Biggs Institute for Alzheimer’s & Neurodegenerative Diseases, University of Texas Health Science Center, San Antonio, TX USA; ^9^ Department of Biochemistry and Structural Biology, The University of Texas Health Science Center at San Antonio, San Antonio, TX USA; ^10^ Department of Population Health Sciences, The University of Texas Health Science Center at San Antonio, San Antonio, TX USA

## Abstract

**Background:**

Oral and gut microbiomes have been associated with Alzheimer’s disease and related dementias (ADRD). Although the role of the gut microbiome and gut dysbiosis in ADRD has been extensively studied, research on the oral microbiome is lacking. Moreover, the synergetic contribution of oral and gut microbiomes to ADRD is unexplored. This study aimed to assess the differential patterns of oral and gut microbiomes and their synergetic effects in patients with mild cognitive impairment (MCI) compared to normal cognition (NC).

**Method:**

Gut and saliva microbiome abundance and diversity measurements were obtained using 16S rRNA gene sequencing of stool and saliva samples from 27 participants (12 MCI, 15 NC, %F = 66.7, Age = 70.2 ± 6.4) recruited in San Antonio, Texas, USA (**Table 1**). The indexes Chao1, ACE, Observe, Shannon, and Simpson were computed to assess the 〈‐diversity of samples. However, the ®‐diversity was investigated after carrying out the principal coordinates analysis (PCoA) based on Bray‐Curtis distances to visualize group separation in compositional data. We used linear discriminant effect size and differential abundant analysis to identify gut and saliva features statistically different between MCI and NC (adjusted p‐value < 0.005).

**Result:**

No differences in bacteria 〈‐ and ®‐diversity between MCI and NC (**Figure 1**) were found. However, we found an increased abundance of oral pathogenic genera, including *Anaeroglobus*, *Centipeda, Cardiobacterium*, *Dialister*, *Fretibacterium*, *Leptotrichia*, *Mycoplasma*, *Tannerella*, and *Treponema* in patients with MCI (**Figure 2**). We also found that MCI patients have a decreased abundance of gut genera *Butyricicoccus*, *Defluviitaleaceae, Lachnospira*, *Paludicola*, *Shuttleworthia*, and *Subdoligranulum*. These differential abundant gut genera have been shown to harbor anti‐inflammatory properties. We did not find evidence of synergetic contributions of oral and gut genera to MCI.

**Conclusion:**

Our results suggest that the saliva microbiome, like the gut microbiome, is disrupted as patients progress from NC to MCI. The pathogenic oral microbes we found were previously linked to periodontal and gingivitis pathogens. Further studies with larger sample sizes are needed to validate these findings.